# The Impacts of Waterproof Insulated Jackets on Lamb Performance on a UK Lowland Farm

**DOI:** 10.3390/ani11010217

**Published:** 2021-01-17

**Authors:** Eleanor Brooke Collins, Nicola Blackie

**Affiliations:** Department Pathobiology and Production Sciences, Animal Welfare Science and Ethics, Royal Veterinary College, Hawkshead Campus, Hawkshead Lane, Hatfield, Hertfordshire AL9 7TA, UK; ecollins7@rvc.ac.uk

**Keywords:** lamb, jackets, hypothermia, sheep, husbandry, production

## Abstract

**Simple Summary:**

Cold stress is a major cause of mortality and morbidity in lambs, with negative implications on both lamb welfare and economics. A recent anecdotal trend has been the provision of insulating jackets to young stock on cattle farms, despite a lack of evidence for their effectiveness in the literature. This study aimed to document the use of similar insulating jackets in lambs to determine if they could reduce the effect of cold stress on lamb growth. Insulating lamb jackets were assessed on their effect on physiological parameters such as temperature and weight gain to determine if they provided an advantage to lamb production. This study found that the jackets had no significant effect on lamb average weight gain, live weight or estimated body temperature. However, jacketed lambs had significantly higher surface temperatures throughout the study.

**Abstract:**

The majority of lamb losses occur within the first two weeks of life, with cold stress being a major cause of lamb morbidity and mortality. This study investigated the effect of insulating lamb jackets on newborn lambs. One hundred and four newborn lambs were randomly allocated by birth date to two treatment groups, (a) non-jacketed (*n* = 52) or (b) jacketed (*n* = 52), for fourteen days after birth. The live weights of lambs were recorded regularly up to 21 days, and average daily weight gains were calculated from these data. For the first two days after recruitment to the study, surface and body temperatures of lambs were also recorded. The jackets significantly increased the lambs’ surface temperatures, providing a warmer microclimate and reduced cold stress for jacketed lambs. There was no significant effect of the insulating jackets on estimated body temperatures, live weights or average daily weight gain of the lambs in this study. There were no detrimental effects of the jackets, and no rejection of lambs occurred.

## 1. Introduction

Nearly 50% of lamb losses between scanning and weaning are due to death within the first 48 hours after birth, and a further 11% of lamb deaths occur between two and fourteen days of age [1]. At an estimated value of £20–£25 per lamb at the time of lambing [1], the first fortnight of a lamb’s life therefore presents a significant potential for improving newborn lamb welfare and associated economic gain.

Hypothermia is a major cause of lamb morbidity and mortality [2]. The rate of change in body temperature of a lamb is a function of the rate of metabolic heat production and the net rate of heat exchange between the animal and its environment. Whilst starvation is the major cause of hypothermia in lambs over 12 hours old, hypothermia in the first few hours of life is primarily caused by excessive heat loss [3].

Thermoregulation is therefore extremely important in newborn lambs. Half of a lamb’s total heat loss occurs through the skin by radiation and conduction [4]. The rate of heat exchange between the skin and the environment is a function of surface area-to-volume ratio, such that the rate of exchange is greater in smaller lambs and increased cold resistance can be found in heavier lambs [5]. Litter size is an important determinant of resistance to cold stress [6], largely due to the smaller birth weight of triplets and twins, which may not be licked dry as quickly [3]. Wet lambs have been shown to become hypothermic in ambient temperatures as high as 15 °C whilst dry lambs are unlikely to become hypothermic unless temperatures are well below freezing point [7]. Exposure to both wind and wetness increases heat loss, with a greater effect in younger lambs [8].

At ambient temperatures outside of the lamb’s thermoneutral zone (TNZ), the lamb must increase its metabolic rate and act to reduce heat loss to maintain thermal balance. The summit metabolic rate (SMR) is an estimation of a lamb’s ability to maintain its body temperature (39–40 °C) in conditions of high heat loss [9]. The SMR can only be sustained for a short period of time, and is achieved when the ambient temperature falls below the lowest critical temperature (LCT) [3]. The lamb can utilise two thermoregulatory mechanisms to increase heat production: shivering thermogenesis and non-shivering thermogenesis. Shivering thermogenesis produces heat through the involuntary movement of skeletal muscle whilst non-shivering thermogenesis utilises brown adipose tissue (BAT) laid down in utero [9]. The relative contribution of these additional mechanisms to SMR changes with age [4]. In neonatal lambs, non-shivering thermogenesis predominates [10,11]. Impaired BAT thermogenesis and the onset of shivering thermogenesis at an earlier age are correlated to poorer control of body temperature and a greater risk of hypothermic death [12,13].

Thermogenesis requires energy from the metabolism of substrates; once a lamb has depleted substrate stores laid down in BAT in utero, it must rely on exogenous intake of these substrates. The starvation of a newborn lamb accelerates the exhaustion of stores and subsequent death as a result of hypothermia and hypoglycaemia [14]. Newborn lambs must therefore display key behaviours to ensure mother acceptance and adequate colostrum and milk intake (vitality), and the ewe must behave to allow this [15]. Indeed, the surface temperature of lambs has been linked to lamb vitality and ewe mothering instinct [16].

Aiding a lamb in maintaining its thermal balance therefore provides an opportunity to improve welfare and enhance production. Thermal balance can be achieved through maintaining the ambient temperature within the lamb TNZ, estimated to be 15–25 °C [17,18] and minimising exposure to environmental conditions that reduce cold resistance. This could be done through the manipulation of a lamb’s environment through the provision of an artificial insulating layer. Despite extensive use of thermal jackets and rugs in companion animals as protection from exposure and cold stress, insulating lamb jackets are not widely used. Increased insulation from a greater fleece depth has been correlated to reduced heat loss in lambs [19], with clipping of the fleece shown to reduce cold resistance in lambs [20]. An insulating cover provided an average of 25% savings in heat loss at an ambient temperature of 5.4–14.6 °C when used on a model lamb [21]. Protection of lambs from cold stress using lamb jackets has also been shown to improve plasma antioxidant defence [22].

Investigations into the effect of jacket usage on productivity in dairy calves showed higher surface and rectal temperatures in calves with jackets than in those without jackets [23,24,25]. Conclusions were drawn that the calf jackets create a microenvironment for the calf, and can act as a barrier to adverse ambient conditions [24], although this did not translate into significant effects on calf health or performance [23,24,25].

No similar field studies have yet quantified the effect of insulating jackets on lamb performance parameters. Thus, the aims of the present study were to (a) determine the efficiency of insulating lamb jackets in creating a microenvironment that minimises heat loss in the lamb; (b) investigate the effect of providing insulating lamb jackets on lamb live weight (LW) and average daily gain (ADG), and; (c) to identify potential guidelines for the use of insulating lamb jackets to maximise the benefit to lamb and farmer.

## 2. Materials and Methods

### 2.1. Animals and Housing

The study was approved by the Clinical Ethical Review Board at the Royal Veterinary College (CR2019-039-2).

One hundred and four twin lambs from sixty ewes lambed at Bolton’s Park Farm (EN6 1NB) between 17 February 2020 and 30 March 2020 were enrolled in the study at between two and four hours old. All lambs used were twins to control for the effect of litter size [7], and because cooling is more pronounced in twins [5]. Recruitment was dependent on an unassisted birth, and on both twins having drunk colostrum and having been dried by the ewe without human intervention. These variables were controlled for due to their known effects on cold resistance in lambs [6,7,12,14,15,26]. Lambs were born to Suffolk or Charolais cross ewes that had been put to either Suffolk, Charolais or Texel rams.

An indoor lambing housing system was used. Ewes were kept in group pens and moved into individual pens upon the birth of the second twin. Individual pens (1.5 m^2^) were located in a large three-sided wooden barn with ventilation slats at six feet along the long sides. At between two and three days old, lambs were tagged, and lambs and ewes turned out into a larger pen in a polytunnel (plastic-covered semi-circular metal-frame building). The larger pens (approximately 6 m × 8 m containing 15 ewes and 30 lambs) contained a mixed group of twin lambs and their ewes from both J (jacketed lambs) and NJ (non-jacketed lambs) groups, separated from other larger pens according to age. In both individual and larger pens, straw bedding was used. The bedding was fully replaced after every use in the individual pens. In the larger pens, fresh straw was bedded down on top of old straw every two to three days. Once in the larger pen, lambs had ad libitum access to hay and fresh water.

### 2.2. Experimental Design

**Figure 1:**
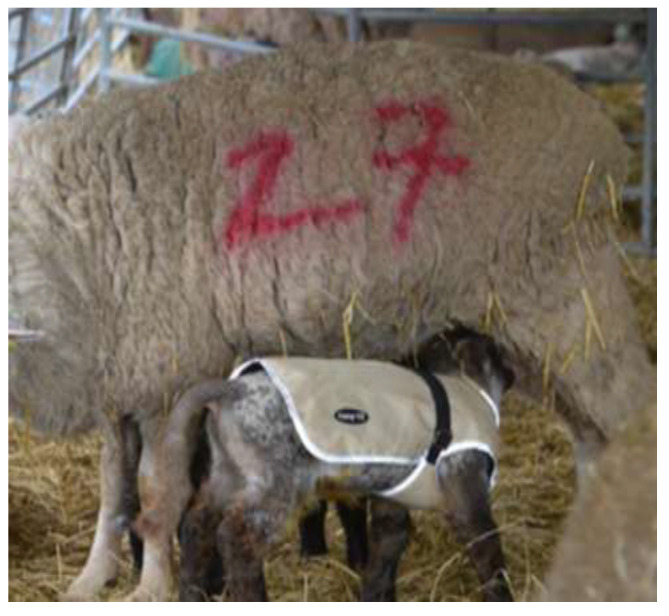
Lamb feeding while wearing the jacket (newborn).

**Figure 2:**
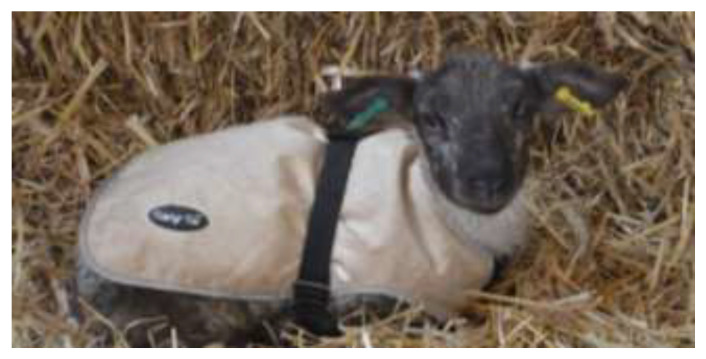
Lamb in jacket showing the fastening strap.

**Figure 3:**
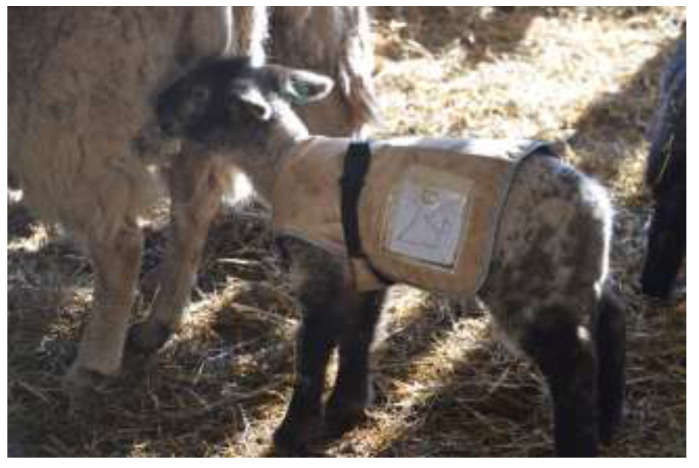
Lamb in jacket approaching two weeks of age; note the jackets appear short at that age but still functional.

On enrolment to the study (time (*t*) = 0), sets of twin lambs (i.e. whole litter) were allocated alternately in birth order in two equal groups (*n* = 52): jacketed (J) (Figure 1, Figure 2 and Figure 3) and non-jacketed (NJ). Jackets were waterproof with an outer shell made of 600D Oxford, 100 g filling and a nylon lining (Cosy Calf, Dorset, UK). The jackets were secured using the strap under the front legs shown in Figure 1, Figure 2 and Figure 3. All jackets were sized “medium”.

First recordings were made prior to jacket placement (time (*t*) = 0). Sets of twins were allocated to groups alternately to balance time of lambing across groups. Lambs born to the same ewe were kept allocated to the same group to reduce risk of rejection of one lamb by the mother. Lambs were also balanced across groups for birth weight, with the average birth weight being 4.82 ± 0.76 kg (Mean ± SD) for group J and 4.89 ± 0.59 kg for group NJ. Jackets were removed from group J after 14 days.

### 2.3. Measurements

Live weights (LWs) were recorded at 0 hours, day 2, day 7 and day 14, as close as possible to exact from birth time. Weight was recorded using a digital spring balance (No More Excess, ASIN: B001E49688), with the lamb suspended in a bag. The balance was zeroed between each recording. A subset of each group (*n* = 15) had weights recorded at day 21 (the rest of the lambs had been moved by the farmer off site). The average daily gain (ADG) was obtained by dividing the difference between consecutive LW measurements by the number of days between the two assessments.

Surface temperature (ST) was recorded at 0 h, 12 h, 24 h and 48 h. The temperature was recorded using an infrared thermometer (Decdeal, ASIN: B076HM96MS; precision 0.1 °C) placed 5 cm from the skin at the spinous process of the scapula. This anatomical position was chosen as (a) it was covered by the jacket; (b) blood flow to the trunk skin is not affected by cold exposure [11] and (c) it is least likely to be affected by the lying position. Where the lamb was observed lying on one side before measurement, the measurement was recorded on the opposite side.

Body temperature (BT) was recorded at 0 h, 12 h, 24 h and 48 h. The temperature was recorded using an infrared thermometer pointed at the anus to give estimated body temperature (Decdeal, ASIN: B076HM96MS).

Ambient temperature (AT) was recorded by taking the minimum and maximum temperature measured between 08:00 h and 20:00 h, and the minimum and maximum temperature measured between 20:00 h and 08:00 h. The ambient temperature was measured using a thermometer placed in the middle of the individual pen barn, situated approximately 1 m above pen height. Data were recorded for the first twelve days of the study, as this was when all the lambs had been moved out of the individual pens and into the polytunnels. All measurements were made by one researcher (EC).

### 2.4. Statistical Analysis

All data were analysed using IBM SPSS Statistics for Windows, version 26 (IBM Corp., Armonk, NY, USA). Outliers were identified using the ROUT test and assessed to determine whether they were anomalous. True anomalies were removed from the data set. D’Agostine and Pearson tests were then used to assess each data column for normality.

These data where then analysed within a linear mixed effects model. Statistical significance of differences in the mean live weight, average daily weight gain, mean surface temperature and mean body temperature between the J and NJ groups were assessed. The effect of jacketing, time, sex, breed and the interaction jacketing x time were included as fixed effects in the model, and lamb and litter were included as random effects. Type I error rate was set at 5%.

## 3. Results

### 3.1. Live Weight and Daily Weight Gain

Live weight increased with age for both the J and NJ groups. Groups were balanced for birth weight upon enrolment of lambs into the study, such that mean birth weights were 4.89 ± 0.59 kg and 4.82 ± 0.76 kg for the NJ and J groups, respectively. There was no effect of the insulating jacket on the LW of lambs at day 2, day 7, day 14 or day 21 (*p* = 0.743). Despite a lower mean birth weight, the J group continued to have a larger LW than the NJ group at all recorded points (Table 1). Despite the lack of significant differences in LW, the difference between the J and NJ groups increased with lamb age, rising from 0.05 kg (SED = 0.217) at day 2 to 0.44 kg (SED = 0.344) at day 21, being favourable for J lambs.

ADG in both the NJ and J groups showed a similar overall pattern (Table 1), with the largest gain being seen between birth and day 2 (NJ: mean = 0.29 kg/day; J: 0.35 kg/day), and a decreasing ADG over time. The difference in the ADG between groups was greatest at day 2, but differences did not reach significance (*p* = 0.496), indicating that there was no effect of the insulating jackets on the ADG of lambs.

### 3.2. Surface and Estimated Body Temperature

At the point of enrolment to the study, ST did not differ between lambs in the NJ and J groups. There was an effect of the insulating jacket on the ST of lambs at all other measurement times, and group J had greater ST at all times with a difference of 3.36 °C (SED = 0.42, *p* ≤ 0.001), 4.24 °C (SED = 0.415, *p* ≤ 0.001) and 4.30 °C (SED = 0.415, *p* ≤ 0.001) at 12, 24 and 48 h respectively.

There was no effect of the insulating jackets on BT at any time point (*p =* 0.661) (Table 2).

### 3.3. Ambient Temperature

**Figure 4:**
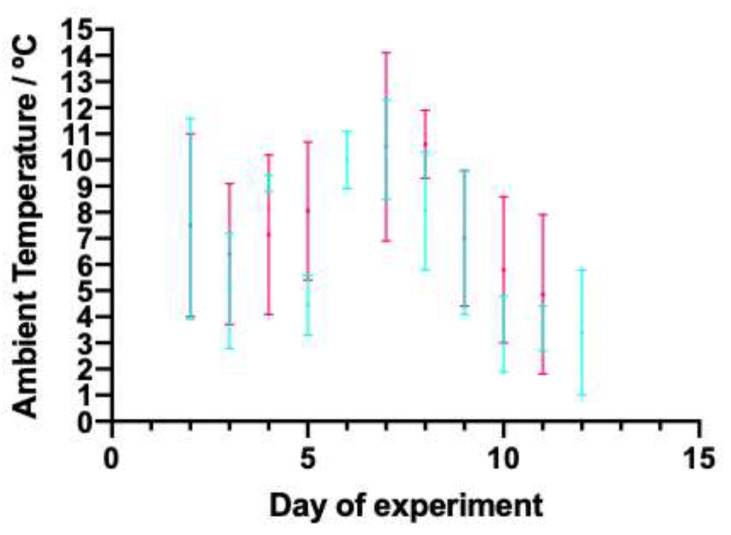
Range of ambient temperature (°C) of the barn that housed the lambs for the first 12 days of the study. Blue lines show the minimum and maximum temperatures reached between 20:00 and 08:00, and pink lines show the minimum and maximum temperatures reached between 08:00 and 20:00. By day 12 of the study, all lambs had moved out of this barn.

The temperature remained below 15 °C throughout the experiment, as shown in Figure 4, with a minimum recorded temperature of 1 °C. The range of ambient temperature was larger during the day than the night for seven of the twelve days during which the temperature was recorded.

## 4. Discussion

The aim of this study was to investigate the effects of lamb jackets on the growth and temperature of lambs in an indoor lambing situation. There were no rejections of lambs or mortality seen during the study.

The ambient temperature throughout this study remained at the lower end of the lambs’ TNZ [17], with all births occurring at temperatures known to result in a more rapid decline in lamb rectal temperature and therefore increased risk of hypothermia than would be seen in lambs born at higher ambient temperatures [27,28]. However, the body temperature of all lambs in the current study remained in the normal range of 39–40 °C [9], suggesting that no lamb achieved SMR during this experiment. This finding is consistent with other studies that have suggested that dry newborn lambs are able to maintain a normal rectal temperature at ambient temperatures below −15 °C [9].

In ambient temperatures outside of the TNZ, lambs must increase their metabolic rate to maintain thermal balance [9]. In the present study, there was a significantly higher ST in the J group compared to the NJ group for all measurements recorded after jackets had been put on. This outcome is consistent with similar studies on dairy calves that have reported significantly greater ST of jacketed calves [24,25]. This result indicates that the jackets are effective in creating a warmer microenvironment for the lambs and are effective insulators.

The reduction in the temperature gradient between the lamb and its immediate environment due to jacket provision should reduce heat loss through the skin—the route by which half of a lamb’s heat is lost [4]. This prediction is supported by a study that quantified heat loss from a model lamb, and showed that an insulating cover reduced heat loss by 25% [21].

To counteract heat loss, two forms of thermogenesis are available to lambs: non-shivering and shivering. In the early days of life, non-shivering thermogenesis is the predominant form of heat production in lambs. The neonatal lamb has a fixed energy availability for non-shivering thermogenesis, as BAT is laid down in utero [9,11]. At colder ambient temperatures, such as those experienced by the NJ group of lambs, BAT is consumed more rapidly, and shivering thermogenesis is recruited at an earlier age. The use of shivering thermogenesis at an earlier age is correlated to poorer control of body temperature and a greater risk of hypothermic death [12,13]. Energy used for shivering thermogenesis is obtained from feed intake [17]. Since lambs will partition available energy between thermoregulation and growth [29], when heat loss is lowered there is an increased net energy gain and thus an increased protein efficiency ratio and average daily growth [18].

In this study, the ST was consistently lower in the NJ group, and as such, these lambs would have had to have increased energy intake to maintain thermogenesis and a competitive growth rate. In the present study, lambs were able to suckle ad libitum from the ewe, and so any increased energy intake in NJ lambs was not quantified. A study that controlled for dietary input examined the effect of temperature on growth rates in hand-reared lambs: lambs raised in a colder environment had a lower mean growth rate throughout the first month of life [29]. Increased feed intake in colder animals has been shown in cattle, with non-jacketed calves having more unrewarded visits to the milk feeder when compared to jacketed calves over the first three weeks of life [25]. These findings suggest that restriction in nutritional intake would have more impact than the microclimate created by the jackets and could explain the lack of significant difference in ADG between the NJ and J lamb groups. 

This investigation into lamb jacket usage was limited by the conditions under which the jackets were tested. The present study was carried out in an indoor lambing system during a mild February when the ambient temperature remained above 1 °C. These environmental conditions and this management system did not present a significant cold risk to lambs, as evidenced by no lamb achieving SMR or a fall in estimated BT outside of normal range during the experiment. Furthermore, lambs were only recruited into the study when they were dry and had drunk colostrum (both wetness and lack of colostrum are known to significantly reduce cold resistance in lambs).

In a system where cold stress is amplified due to ambient temperatures being below LCT, or due to increased exposure to wetness or wind [30], the benefit of jacket provision might be more significant. The jackets may also provide an increased benefit to individual lambs with a lower degree of cold resistance, determined by factors such as birth weight, litter size, colostrum intake and key behavioural ewe and lamb traits [11,15]. This indicates that further field studies are required to assess the effect of lamb jackets in conditions more stressful than those of this study in order to determine guidelines for jacket usage.

## 5. Conclusions

Provision of an insulating jacket increased the surface temperature experienced by lambs. Under the environmental and management conditions of the present study, the jackets had no significant effect on markers of production such as live weight or average daily weight gain. The authors suggest that under more extreme conditions of increased cold stress, jackets could offer the potential to provide welfare and economic benefits to lambs.

## Figures and Tables

**Table 1:** Mean live weight (LW; kg) and average daily gain (ADG; kg/d) of non-jacketed (NJ) and jacketed (J) lambs for the first 21 days of life.

Parameter	Treatment	Day						*p*-Value		
		0	2	7	14	21	SE	Day	Jacket	Day × Jacket
LW (kg)	NJ	4.89	5.47	6.70	8.13	8.76	0.205	<0.001	0.743	0.528
	J	4.82	5.52	6.78	8.21	9.20				
ADG (kg/d) ^a^	NJ	-	0.29	0.25	0.20	0.19	0.018	<0.001	0.496	0.123
	J		0.35	0.25	0.21	0.17				

^a^ ADG from a previous time point to the day indicated. SE indicates standard error.

**Table 2:** Mean surface temperature (°C) and estimated body temperature (°C) of non-jacketed (NJ) and jacketed (J) lambs for the first 48 h of life.

Parameter	Treatment	Hours after Birth						*p*-Value	
		0	12	24	48	SE	Time	Jacket	Time × Jacket
Surface Temperature (°C)	NJ	23.28	24.73	23.86	22.74	0.201	<0.001	<0.001	<0.001
	J	22.94	28.09	28.10	27.04				
Estimated Body Temperature (°C)	NJ	38.48	39.54	39.67	39.75	0.256	<0.001	0.661	0.270
	J	38.46	39.12	39.66	40.02				

SE indicates standard error.

## Data Availability

The datasets used and analyzed during the current study are available from the corresponding author on reasonable request.
